# Calreticulin expression in relation to exchangeable Ca^2+^ level that changes dynamically during anthesis, progamic phase, and double fertilization in *Petunia*

**DOI:** 10.1007/s00425-014-2178-z

**Published:** 2014-09-28

**Authors:** Robert Lenartowski, Anna Suwińska, Marta Lenartowska

**Affiliations:** 1 Laboratory of Isotope and Instrumental Analysis, Faculty of Biology and Environment Protection, Nicolaus Copernicus University, Toruń, Poland; 2 Laboratory of Developmental Biology, Faculty of Biology and Environment Protection, Nicolaus Copernicus University, Toruń, Poland

**Keywords:** Calcium homeostasis, Chaperone activity, Embryo sac, Nucleus, Pollen germination and tube growth, Receptive synergid, Stigma

## Abstract

Calcium (Ca^2+^) plays essential roles in plant sexual reproduction, but the sites and the mechanism of Ca^2+^ mobile storage during pollen–pistil interactions have not been fully defined. Because the Ca^2+^-buffering protein calreticulin (CRT) is able to bind and sequester Ca^2+^, it can serve as a mobile intracellular store of easily releasable Ca^2+^ and control its local concentration within the cytoplasm. Our previous studies showed an enhanced expression of *Petunia hybrida CRT* gene (*PhCRT*) during pistil transmitting tract maturation, pollen germination and tube outgrowth on the stigma, gamete fusion, and early embryogenesis. Here, we demonstrate that elevated expression of CRT results in the accumulation of this protein in response to anthesis, pollination, sperm cells deposition within the receptive synergid and fertilization, when the level of exchangeable Ca^2+^ changes dynamically. CRT localizes mainly to the endoplasmic reticulum and Golgi compartments in the pistil transmitting tract cells, germinated pollen/tubes, and sporophytic/gametophytic cells of the ovule and corresponds with loosely bound Ca^2+^. Additionally, the immunogold research shows, for the first time, highly selective CRT distribution in specific nuclear sub-domains. On the basis of our results, we discuss the possible functions of CRT with respect to the critical role of Ca^2+^ homeostasis during key events of the multi-step process of generative reproduction in angiosperms.

## Introduction

In flowering plants, Ca^2+^ is a critical regulator of pollen germination and recognition, directional growth of the pollen tube to the ovule, gamete fusion, and activation of the fertilized egg cell (see reviews by Ge et al. 2007; Hepler et al. 2012; Dresselhaus and Franklin-Tong 2013; Steinhorst and Kudla 2013). Ca^2+^ exists in living cells in three forms, each required for specific activities in cellular metabolism and physiological functions (see review by Ge et al. 2007): (1) insoluble covalently bound Ca^2+^ plays mainly a structural role; (2) cytosolic free Ca^2+^ acts as a second messenger, serving as an early product in multiple signal transduction pathways; and (3) loosely bound Ca^2+^ is typically associated with fixed and mobile anions and is in dynamic equilibrium with the free Ca^2+^. Stored Ca^2+^ is sequestered in different cell compartments (such as the cell wall) and specific organelles (such as the endoplasmic reticulum(ER)) where it can be associated with specific proteins that buffer and release Ca^2+^ to control its local concentration. This pool of Ca^2+^ is exchangeable and can transform into the other forms when and where it is needed. Different Ca^2+^ forms function in interactions of the male gametophyte (pollen grain and its structural component, the pollen tube) with the female sporophytic tissues (pistil transmitting tract and ovular somatic tissues) and female gametophyte (the embryo sac) (see reviews by Ge et al. 2007; Hepler et al. 2012; Dresselhaus and Franklin-Tong 2013; Steinhorst and Kudla 2013). The importance of Ca^2+^ in these processes suggests that Ca^2+^-binding proteins may also play some important roles during complicated communications of the male and female gametophytes in planta. A good candidate for providing this function in flowering plants is CRT. This multifunctional Ca^2+^-binding/buffering protein containing an ER targeting and retention signals, is highly expressed in eukaryotic cells, and has a variety of cellular functions, both inside and outside of the ER lumen (see reviews by Gelebart et al. 2005; Jia et al. 2009; Michalak et al. 2009; Thelin et al. 2011; Qiu et al. 2012a). CRT acts as a Ca^2+^-buffering lectin-like molecular chaperone involved in proper folding, quality control, and maturation of newly synthesized glycoproteins within the secretory pathway. Additionally, CRT plays a role in regulation of Ca^2+^ signaling and homeostasis.

CRTs have been identified in 15 different plant species belonging to gymno- and angiosperms (Menegazzi et al. 1993; Chen et al. 1994; Denecke et al. 1995; Hassan et al. 1995; Kwiatkowski et al. 1995; Napier et al. 1995; Dresselhaus et al. 1996; Lim et al. 1996; Opas et al. 1996; Coughlan et al. 1997; Nelson et al. 1997; Nardi et al. 1998; Navazio et al. 1998; Li and Komatsu 2000; Lenartowska et al. 2002, 2009; Jia et al. 2008). In plants, distinct expression patterns and posttranscriptional modifications of CRT contribute to the ability of this protein to have diverse roles. These include response to biotic and abiotic stresses such as pathogen attacks, cold, drought, salt, and exogenous phytohormones. Plant CRTs are also involved in regulation of complex processes such as gene expression via direct interactions with steroid receptors, nuclear transport, cell adhesion, immunity, regeneration, apoptosis, and cell-to-cell communication by plasmodesmata (see reviews by Jia et al. 2009; Thelin et al. 2011; Qiu et al. 2012a). Besides its main localization in the ER, CRT has been also found outside this compartment in plant cells, including Golgi stacks and secretory granules (Borisjuk et al. 1998; Navazio et al. 2002; Lenartowska et al. 2002, 2009; Nardi et al. 2006), tapetosome vesicles (Hsieh and Huang 2005), the cytosol (Lenartowska et al. 2002; Jia et al. 2008), protein bodies (Torres et al. 2001; Šamaj et al. 2008), nucleus (Denecke et al. 1995; Napier et al. 1995; Lenartowska et al. 2002), plasma membrane and the cell surface (Borisjuk et al. 1998; Lenartowska et al. 2002; Navazio et al. 2002), plasmodesmata (Baluška et al. 1999; Laporte et al. 2003; Chen et al. 2005; Lenartowska et al. 2009; Christensen et al. 2010), and the spindle apparatus of dividing cells (Denecke et al. 1995), indicating that plant CRTs may be involved in multiple cellular processes.

Work from our lab and others has provided evidence that CRT plays a role in plant sexual reproduction. CRT is expressed in flower organs including anthers and pistils (Nelson et al. 1997; Lenartowska et al. 2002, 2009; Nardi et al. 2006) as well as in pollen, pollen tubes, and sperm cells (Williams et al. 1997b; Nardi et al. 1998, 2006; Navazio et al. 1998; Lenartowska et al. 2002, 2009; Qin et al. 2009). Moreover, levels of CRT mRNA or protein are up-regulated during fertilization, embryogenesis, and seed maturation (Chen et al. 1994; Denecke et al. 1995; Dresselhaus et al. 1996; Coughlan et al. 1997; Nelson et al. 1997; Borisjuk et al. 1998; Navazio et al. 2002; Christensen et al. 2010). Given its Ca^2+^-binding/buffering property, CRT could effectively provide transient storage and release of Ca^2+^ during these processes. Our previous characterization of *PhCRT* mRNA expression patterns by Northern blot and in situ hybridization revealed enhanced expression of *PhCRT* during pistil transmitting tract maturation and at the beginning of the progamic phase, when pollen germinates and pollen tubes outgrow on the stigma (Lenartowski et al. 2014). Furthermore, we observed the highest level of *PhCRT* mRNA in the ovary at fertilization and during early embryogenesis. We speculated that these expression patterns indicate that CRT is involved in regulation of Ca^2+^ homeostasis during key events in sexual reproduction of flowering plants. Here, we have further tested this hypothesis by examining sites of intracellular exchangeable Ca^2+^ storage and by determining the level and localization of CRT during anthesis, pollen–pistil interactions, and gamete fusion in *Petunia*.

## Materials and methods

### Plant material

Commercial cultivars of *Petunia hybrida* were grown at room temperature. Whole pistils were dissected from unpollinated flower buds 1 day before anthesis, from unpollinated flowers at anthesis, and from flowers cross-pollinated with compatible pollen at anthesis. To examine stigma receptivity, successive growth of pollen tubes during the progamic phase and fertilization stage, pistils were dissected from unpollinated flowers and from flowers at different time points after pollination. Samples of stigmas, styles, and ovules were prepared according to the standard protocol to obtain semi-thin sections that were stained with 0.1 % methylene blue and observed by light microscopy. For immunoblot analysis, unpollinated and pollinated whole pistils or pistils divided into stigma–style and ovary fragments were used. Selected tissue samples of stigmas and ovules were also prepared for electron microscopy cytochemical and immunocytochemical studies according to the protocols as described below. All experiments were repeated at least three times during several growing seasons with similar results.

### Localization of loosely bound Ca^2+^ by potassium antimonate precipitation

Samples of stigmas and ovules dissected from unpollinated and pollinated pistils were fixed with freshly prepared 2 % (w/v) potassium antimonate, 2 % (v/v) glutaraldehyde, and 2 % (v/v) formaldehyde in 0.1 M phosphate buffer (KH_2_PO_4_, pH 7.8) for 4 h at room temperature, and then subsequently postfixed with 1 % (v/v) osmium tetroxide (OsO_4_) in the same buffer-antimonate solution for 12 h at 4 °C. Next, samples were dehydrated in graduated ethanol concentrations and embedded in Poly/Bed 812 resin (Polysciences) according to the standard protocol. Ultra-thin longitudinal sections were collected on copper grids, stained with 2.5 % (w/v) uranyl acetate and 0.4 % (w/v) lead citrate solutions, and examined by transmission electron microscopy (Jeol EM 1010) at 80 kV. The presence of Ca^2+^ in the precipitates was confirmed previously using energy-dispersive X-ray microanalysis (Lenartowska et al. 1997; Bednarska et al. 2005).

### Western blot analysis

100 mg of whole *Petunia* pistils or pistils divided into stigma–style fragments and ovaries were dissected from unpollinated and pollinated flowers, frozen in liquid nitrogen, and stored at −80 °C until they were used. They were then homogenized in liquid nitrogen, and soluble proteins were extracted in 50 mM Tris–HCl (pH 7.5), 1 mM EGTA, 2 mM DTT plus 1 mM PMSF and cOmplete Protease Inhibitor Cocktail (Roche) according to the manufacturer’s recommendation. The homogenates were centrifuged at 16,000*g* for 30 min at 4 °C. Protein concentrations of the supernatants were measured with the Bio-Rad DC Protein Assay according to the manufacturer’s instructions. Equal amounts of proteins were separated by electrophoresis on a 12.0 % SDS–PAGE gel and then the proteins were semi-dry transferred to Amersham PVDF Hybond-P membrane (GE Healthcare). Blocked blots were probed with a rabbit polyclonal antibody against maize CRT (CRT PAb) (Napier et al. 1995), washed, and probed with antibody against rabbit IgG conjugated with horseradish peroxidase (HRP, Sigma). Signal was detected with the Amersham ECL Advance Western Blotting Detection Kit according to the manufacturer’s guidelines (GE Healthcare). Membranes were stripped according to the manufacturer’s instruction (GE Healthcare) and re-probed with goat polyclonal antibody against *Arabidopsis* α-tubulin (Santa Cruz Biotechnology) and then with anti-goat IgG-HRP secondary antibody (Santa Cruz Biotechnology). Detection was performed as described above. Each Western blot was performed a minimum of three times for each experiment, and representative blots shown. Quantification of signals was done with Image Gauge 3.4 software (Science Lab99). Statistical significance of data was determined by a one-way ANOVA test.

### Immunogold localization of CRT

The samples of stigmas and ovules dissected from pollinated pistils were fixed with 4 % (v/v) formaldehyde and 0.25 % (v/v) glutaraldehyde in phosphate-buffered saline (PBS, pH 7.2) for 1 h at room temperature (slight vacuum infiltration) followed by overnight fixation at 4 °C. Fixed samples were dehydrated in graduated ethanol concentrations, embedded in LR Gold resin (Fluka) according to the standard protocol, and ultra-thin longitudinal sections were collected on Formvar-coated nickel grids. The sections were then incubated with blocking solution containing 3 % (w/v) bovine serum albumin (BSA) in PBS buffer, pH 7.2, for 5 min at room temperature, incubated in 1:20 dilution of a primary CRT PAb in PBS supplemented with 0.3 % (w/v) BSA for 2 h at room temperature, and then incubated with gold-conjugated goat anti-rabbit IgG antibody (BBInternational), diluted 1:100 in PBS buffer with 0.2 % (w/v) BSA for 1.5 h at room temperature. In the control, incubation with the CRT PAb was omitted. Finally, the sections were stained with 2.5 % (w/v) uranyl acetate and 0.4 % (w/v) lead citrate solutions and examined by transmission electron microscopy as above.

## Results

**Fig. 1:**
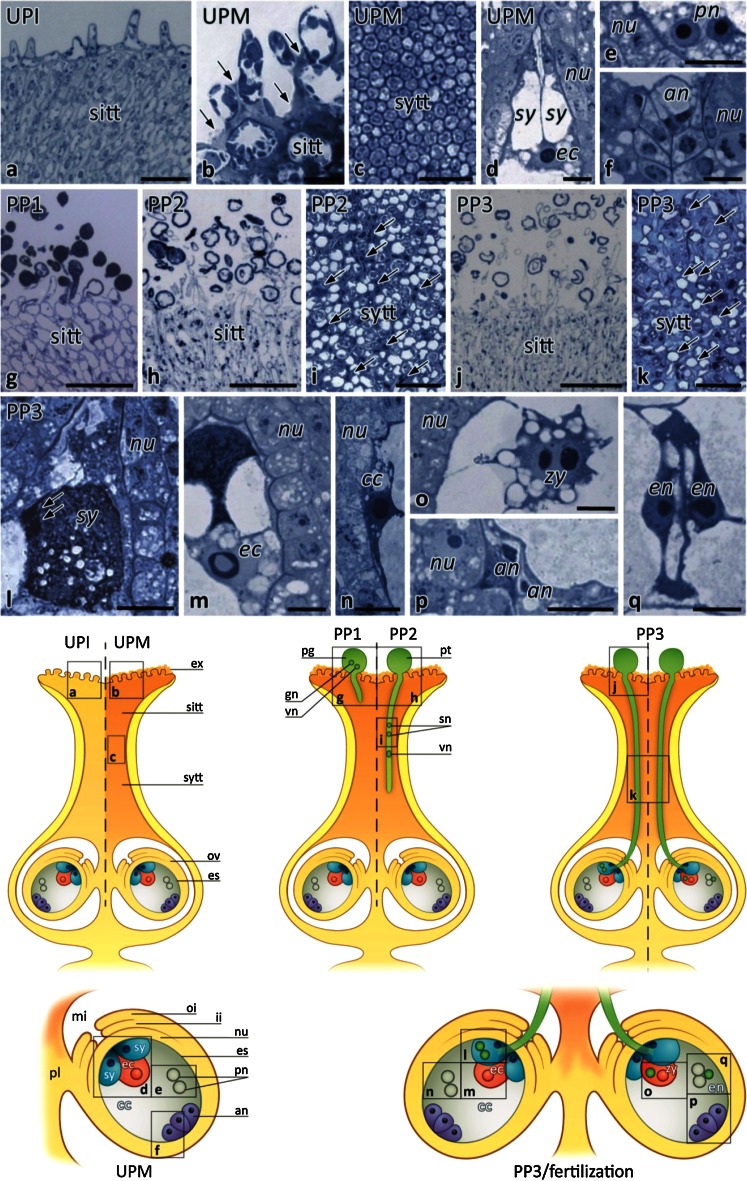
Methylene blue staining of *Petunia* stigmas, styles, and ovules before anthesis, at anthesis, during the progamic phase, and fertilization. **a** Longitudinal section of the stigma dissected from a flower bud (UPI pistil). **b** Longitudinal section of the stigma at anthesis (UPM pistil); in the stigma transmitting tract (*sitt*) at the receptive stage, the exudate fills intercellular spaces between the sitt cells and covers the stigma surface (*arrows*). **c** Cross section of the style transmitting tract (*sytt*) before pollination. **d–f** Longitudinal sections of the ovule dissected from the UPM pistil; sister synergids (*sy*) and the egg cell (*ec*) are located at the micropylar pole of the embryo sac (**d**), while the polar nuclei (*pn*) of the central cell and the antipodals (*an*) are located at the chalazal pole of the embryo sac (**e** and **f**, respectively). Analogous positions of these cells within the female gametophyte were observed after pollination during the first (PP1) and second (PP2) stages of the progamic phase (data not shown). **g**, **h** Longitudinal sections of the pollinated stigmas during the PP1 (**g**) and PP2 (**h**) stages; at the PP1 stage, pollen germinates and pollen tubes outgrow within the sitt, whereas at the PP2 stage, many germinated pollen grains are devoid of cytoplasm that has been displaced into the growing tubes. The sytt of the pistil during the PP1 stage is devoid of pollen tubes that penetrate sitt during this stage (data not shown). **i** Cross section of the sytt at the PP2 stage; cytoplasmic apical/sub-apical zones of growing pollen tubes are visible between sytt cells (*arrows*). **j**, **k** Longitudinal section of the stigma and cross section of the sytt at the PP3 stage (respectively); both regions of the pistil are fully penetrated by the pollen tubes, and their vacuolized zones are visible between degenerated sytt cells (*arrows* in **k**). **l–q** Longitudinal sections of the ovule at the late stage of the progamic phase (PP3) during pollen tube rupture within the receptive synergid (**l–n**) and at fertilization (**o–q**); the sperm cells are released within the receptive synergid (**l**); the highly active egg cell (*ec*, **m**) and the central cell (*cc*, **n**) are both located at the micropylar pole of the embryo sac just before gamete fusion; a diploid zygote (*zy*, **o**), degenerating antipodals (**p**), and developing endosperm (*en*, **q**) are present within the embryo sac at the fertilization stage. *Cartoons* explain the position of the sections along the pistil. *es* embryo sac, *ex* exudate, *gn* generative nucleus, *ii* inter integument, *mi* micropyle, *nu* nucellus, *oi* outer integument, *ov* ovule, *pg* pollen grain, *pl* placenta, *pt* pollen tube, *sn* sperm nucleus, *vn* vegetative nucleus. *Bars* 100 μm (**a**, **g**, **h**, **j**), 20 μm (**b**, **c**, **i**, **k**), 10 μm (**d–f**, **l–q**)

We previously used aniline blue to characterize the successive growth of the pollen tubes in *Petunia* pistil (Lenartowski et al. 2014). Here, using the semi-thin sections of *Petunia* stigmas, styles and ovules stained with methylene blue, we could detect two main stages before pollination and three main stages after pollination, covering pistil transmitting tract maturation, the progamic phase, and gamete fusion (Fig. 1). The subsequent stages were defined as: (1) the unpollinated immature pistil before anthesis without exudate on the stigma (UPI); (2) the unpollinated mature pistil at anthesis with exudate on the receptive stigma (UPM); (3) the first stage of the progamic phase, when pollen germinate and tubes outgrow on the stigma (PP1); the second stage of the progamic phase, when pollen tubes penetrate the stigma/style transmitting tract (PP2); and the third stage of the progamic phase, when pollen tubes grow into ovules (PP3) and fertilization can occur.

### Distribution of loosely bound Ca^2+^ in stigmas and ovules before and after pollination

The intracellular sites of Ca^2+^ storage during pollen–pistil interactions have not been fully described. Thus, we used potassium antimonate precipitation to compare distribution of exchangeable Ca^2+^ in stigmas and ovules at selected stages before and after pollination.

### Stigmas

**Fig. 2:**
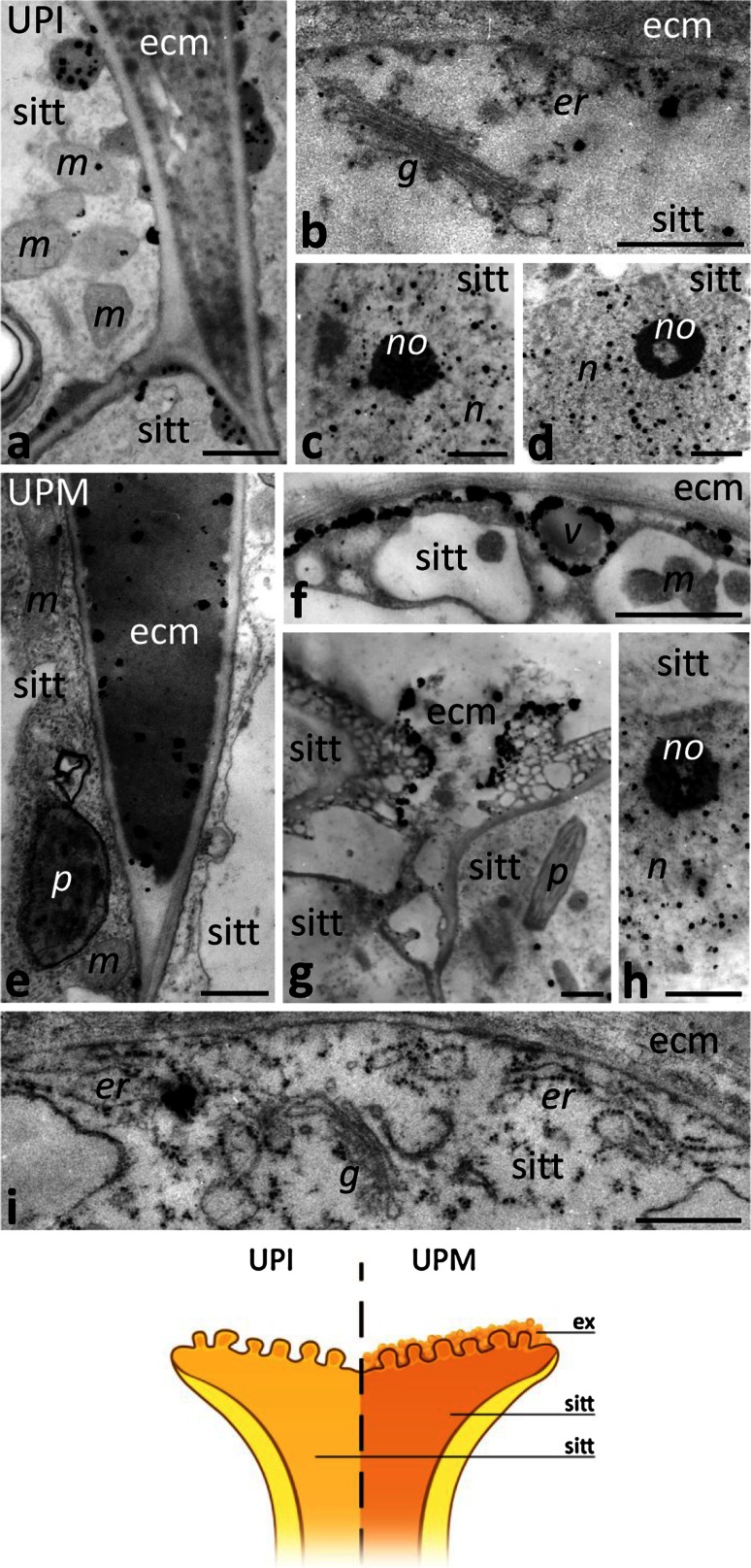
Localization of loosely bound Ca^2+^ by potassium antimonate precipitation in the *Petunia* sitt before and at anthesis. **a–d** Sitt of the UPI pistil before anthesis. **e–i** Sitt of UPM pistil at anthesis. *Cartoon* shows the analyzed regions of the UPI and UPM pistils. *ecm* extracellular matrix, *er* endoplasmic reticulum, *ex* exudate, *g* Golgi stack, *m* mitochondrium, *n* nucleus, *no* nucleolus, *p* plastid, *v* vesicle. *Bars* 1 μm (**a**, **c–h**), 500 nm (**b**, **i**)

In UPI pistil before anthesis, we observed few electron-dense Ca^2+^ precipitates (Ca^2+^ ppts) in the cytoplasm of stigma transmitting tract (sitt) cells (Fig. 2a, b). At this stage, Ca^2+^ ppts were found associated with vesicles under the cell wall (Fig. 2a) as well as in the ER and Golgi stacks (Fig. 2b). At the UPI stage, Ca^2+^ ppts were prevalent in the nuclei and nucleoli of sitt cells (Fig. 2c, d). The *Petunia* stigma is wet, due to secretion at the receptive stage. During anthesis at the UPM stage, exudate is secreted to the extracellular matrix (ecm) of sitt (Fig. 1b, arrows). At this stage, we observed numerous Ca^2+^ ppts in the electron-dense exudate that filled the intercellular spaces between sitt cells (Fig. 2e) and that covered the receptive stigma (Fig. 2g). In the cytoplasm of the sitt cells Ca^2+^ ppts accumulated mainly in the ER cisternae and Golgi stacks (Fig. 2i). They were also associated with some vesicles present in peripheral cytoplasm (Fig. 2f). At anthesis, Ca^2+^ ppts were still prevalent in the nuclei and nucleoli of sitt cells (Fig. 2h).

**Fig. 3:**
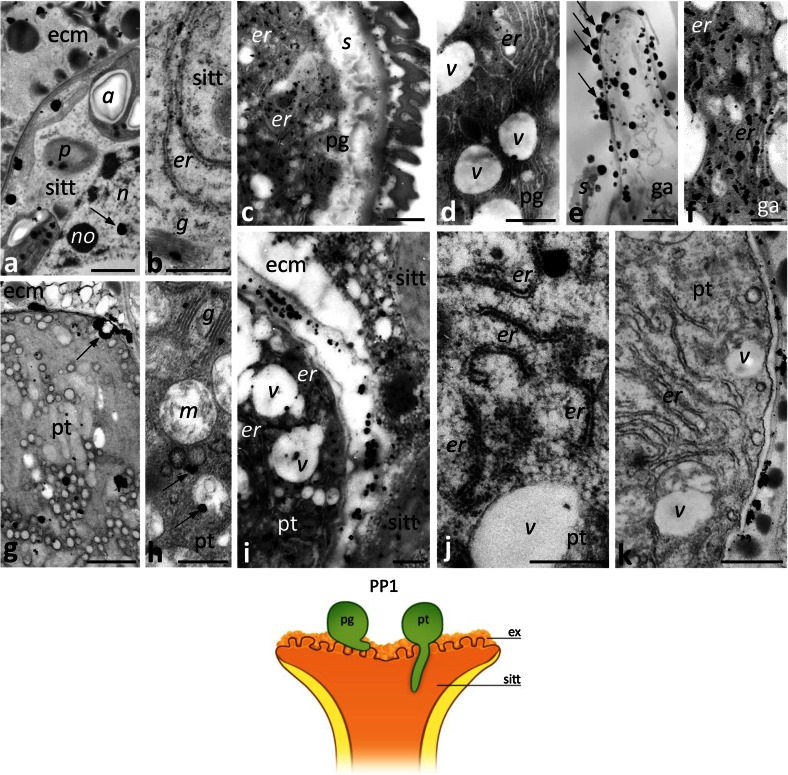
Localization of loosely bound Ca^2+^ by potassium antimonate precipitation in the *Petunia* sitt at the PP1 stage. **a**, **b** The sitt after pollination. **c–f** sections of germinated pollen grain (*pg*) with the germinal aperture (*ga*). **g–k** cross sections of different zones of the growing pollen tube (*pt*); apical zone with numerous vesicles (**g**) and sub-apical zone (**h–k**) containing Golgi stacks (*g*) and ER cisternae (*er*). *Cartoon* shows the analyzed region of the pistil at the PP1 stage. *a* amyloplast, *ecm* extracellular matrix, *ex* exudate, *n* nucleus, *no* nucleolus, *p* plastid, *s* sporoderm, *v* vesicle. *Bars* 1 μm (**a**, **c**, **e**, **g**, **i**), 500 nm (**b**, **d**, **f**, **h**, **j**, **k**)

In the PP1 stage, when pollination starts, there was no obvious change in the level of Ca^2+^ ppts in the stigma ecm or within the cytoplasm of the sitt cells. We detected Ca^2+^ ppts extracellularly in the electron-dense exudate (Fig. 3a) and intracellularly in the ER, and in dictyosomes (Fig. 3b). One clear difference between the UPM and PP1 stages was that there were far fewer Ca^2+^ ppts in the nuclei of sitt cells at the PP1 stage; only single ppts were observed in the chromatin (Fig. 3a, arrow). After pollination, numerous germinated pollen grains and outgrowing tubes were observed on the receptive stigma (Fig. 1g). At the beginning of the progamic phase, pollen tubes penetrate the secretory sitt but do not enter the style transmitting tract (sytt). At this stage, Ca^2+^ ppts most strongly labeled the cytoplasm of germinated pollen, where they were found in the ER (Fig. 3c, d). These ppts were also present within the germinal aperture (Fig. 3e), where they accumulated in the ER cisternae (Fig. 3f). Numerous Ca^2+^ ppts were also found in the sitt ecm, adjacent to the outgrowing apex of the pollen tube (Fig. 3e, arrows). A similar high level of Ca^2+^ ppts was observed in the growing pollen tubes, particularly in their apical and sub-apical zones (Fig. 3g–j). In the tube apex, Ca^2+^ ppts were localized mainly in numerous cytoplasmic vesicles (Fig. 3g) or adjacent to the plasma membrane (Fig. 3g, arrow). In the sub-tip zone of the pollen tube, Ca^2+^ ppts were associated with Golgi stacks (Fig. 3h) and electron-transparent or electron-dense vesicles (Fig. 3h, arrows). We observed the largest number of both small and large Ca^2+^ ppts in the ER, which is the most prominent organelle in the sub-apical zone of the growing tube (Fig. 3i, j). However, the ER cisternae of some pollen tubes were devoid of Ca^2+^ ppts (Fig. 3k). In such tubes, Ca^2+^ ppts were localized preferentially at the cell wall. This result suggests that accumulation of exchangeable Ca^2+^ within the ER may be temporary. At the PP1 stage, a large number of Ca^2+^ ppts was also observed between the growing tube and the degenerating sitt cell, at the zones of local adhesion between the tube and the ecm of the sitt (Fig. 3i). Together, these results indicate that a significant increase of exchangeable Ca^2+^ level occurs during two important events: intensified secretory activity of the maturing sitt at anthesis and pollen germination/outgrowth of pollen tubes on the stigma.

### Ovules

The female gametophyte of *Petunia* develops according to the Polygonum type and consists of two synergids, the egg cell, the central cell containing two polar nuclei, and three antipodals (Fig. 1d–f). During the late stage of the progamic phase (PP3), pollen tube grows along the placenta and reaches the micropylar end of the ovule, where the embryo sac is located. Then the pollen tube directly enters the receptive synergid and releases male gametes, commonly called the sperm cells (Fig. 1l, arrows). One of the sperm cells fuses with the haploid egg cell (Fig. 1m) to generate a diploid zygote/embryo (Fig. 1o), whereas the second one fuses with a diploid central cell (Fig. 1n) to form a nursing tissue, the triploid endosperm (Fig. 1q).

**Fig. 4:**
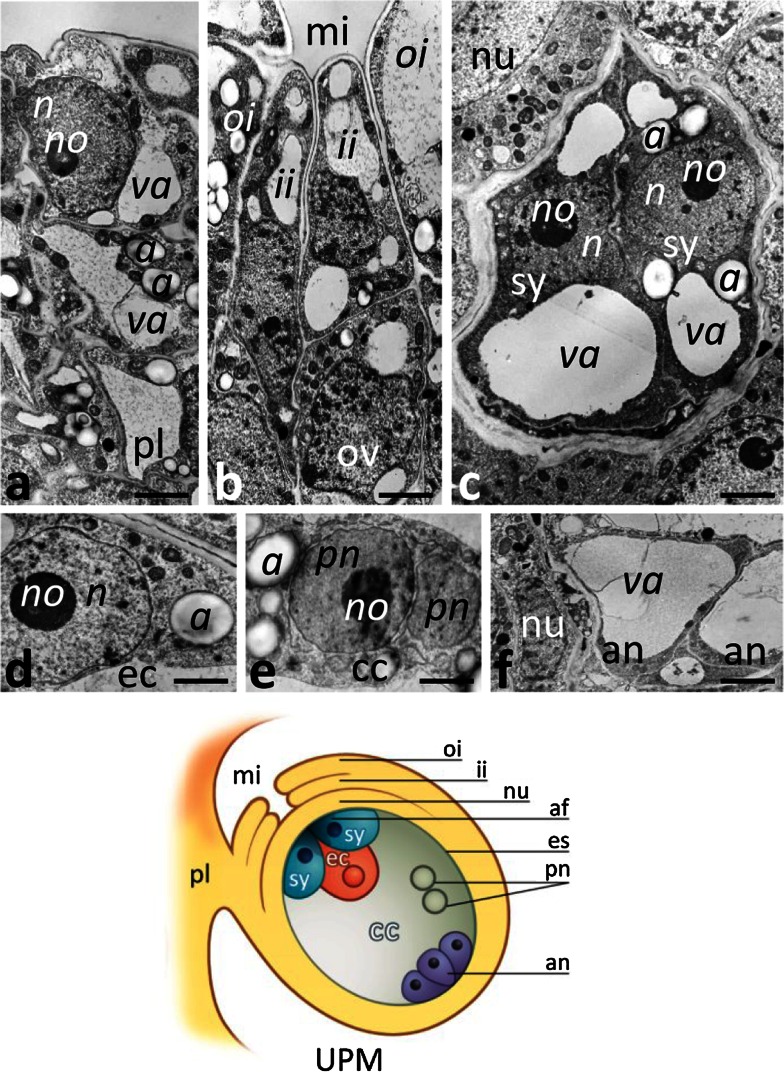
Localization of loosely bound Ca^2+^ by potassium antimonate precipitation in the *Petunia* placental tissue and at the micropylar end of the ovule before pollination. **a** Placenta tissue (*pl*); **b** the micropyle (*mi*) of the ovule (*ov*); **c** sister synergids (*sy*); **d** the egg cell (*ec*); **e** the polar nuclei (*pn*) of the central cell (*cc*); **f** antipodals (*an*). *Cartoon* shows the ovule of the UPM pistil. *a* amyloplast, *af* filiform apparatus, *es* embryo sac, *ii* inter integument, *n* nucleus, *no* nucleolus *nu* nucellus, *oi* outer integument, *va* vacuole. *Bars* 2 μm

**Fig. 5:**
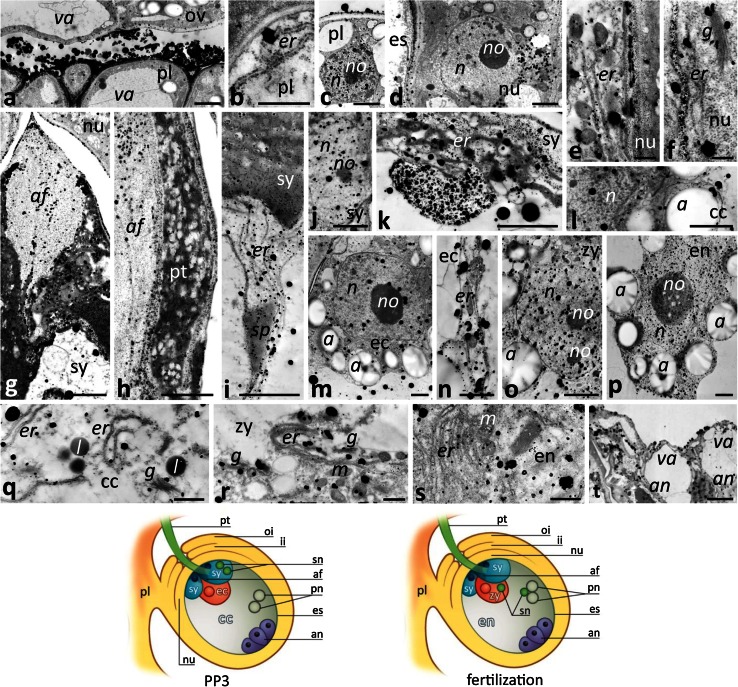
Localization of loosely bound Ca^2+^ by potassium antimonate precipitation in the *Petunia* placental tissue and at the micropylar end of the ovule during the PP3 stage and at fertilization. **a–c** Placenta tissue (*pl*); **d–f** nucellus (*nu*); **g–k** the receptive synergid (*sy*) penetrated by the pollen tube (*pt*); **l**, **q** the central cell (*cc*); **m**, **n** the egg cell (*ec*); **o**, **r** the diploid zygote (*zy*); **p**, **s** the developing endosperm (*en*); **t** degenerating antipodals (*an*). *Cartoons* show the ovules at the PP3 stage during sperm cells deposition within the receptive synergid and at fertilization. *a* amyloplast, *af* filiform apparatus, *er* endoplasmic reticulum, *es* embryo sac, *g* Golgi stack, *ii* inter integument, *l* lipid drop, *m* mitochondrium, *n* nucleus, *no* nucleolus, *oi* outer integument, *ov* ovule, *pn* polar nuclei, *sn* sperm nucleus, *sp* sperm cell, *va* vacuole. *Bars* 2 μm (**a**, **c**, **d**, **g**, **m**, **o–p**, **t**), 1 μm (**n**), 500 nm (**b**, **e**, **f**, **q–s**)

Before pollination, at the UPM stage, we did not observe numerous Ca^2+^ ppts in the placenta (Fig. 4a), somatic cells of the ovule micropylar end (Fig. 4b), and inside the embryo sac, including the sister synergids (Fig. 4c), the egg cell (Fig. 4d), the central cell (Fig. 4e), or the antipodals (Fig. 4f). Instead, during the PP3 stage, when pollen tubes grew into the receptive synergid and fertilization occurred, a drastic increase of Ca^2+^ ppts was observed both in the placenta and in the ovule (Fig. 5). Ca^2+^ ppts were evident extracellularly between the placenta surface and the micropylar region of the ovule (Fig. 5a). Inside the placenta cells, numerous Ca^2+^ ppts were localized in the ER (Fig. 5b) and nucleus (Fig. 5c). We also observed Ca^2+^ ppts in nucellus cells, particularly at the micropylar end of the ovule. They were prominent in the nucellus cell cytoplasm (Fig. 5d), including the ER (Fig. 5e, f) and Golgi stacks (Fig. 5f), and also in the nucleus (Fig. 5d). However, the highest concentration of Ca^2+^ ppts was in the receptive synergid, both in the cytoplasm and in the filiform apparatus (Fig. 5g), a structure along which the pollen tube elongates (Fig. 5h). Numerous Ca^2+^ ppts were also found in the ER surrounding the sperm cells released in the synergid (Fig. 5i) and in the synergid nucleus (Fig. 5j). Within the receptive synergid, an extremely large mass of Ca^2+^ ppts was observed in the cytoplasm near the location of sperm cells deposition. In this area, Ca^2+^ ppts were localized in the ER and in large patches near this cell compartment (Fig. 5k). Ca^2+^ ppts were also common in the egg cell (Fig. 5m, n) and in the central cell (Fig. 5l, q). In these cells, Ca^2+^ ppts were present both in the nuclei and cytoplasmic organelles such as the ER and dictyosomes. The level of Ca^2+^ ppts was higher in the zygote and endosperm after fertilization (Fig. 5o, p, respectively); at this stage, numerous Ca^2+^ ppts were evident in the nuclei and nucleoli and within the cytoplasm, where numerous ER cisternae and Golgi stacks were present (Fig. 5r, s). The lowest concentration of Ca^2+^ ppts was in the antipodals located at the chalazal end of the embryo sac (Fig. 5t). Overall, our data indicate that the level of exchangeable Ca^2+^ increases substantially during the processes of pollen tube entry in the receptive synergid, gamete fusion, and early embryogenesis.

### CRT expression profiles before pollination, during the progamic phase, and at fertilization

**Fig. 6:**
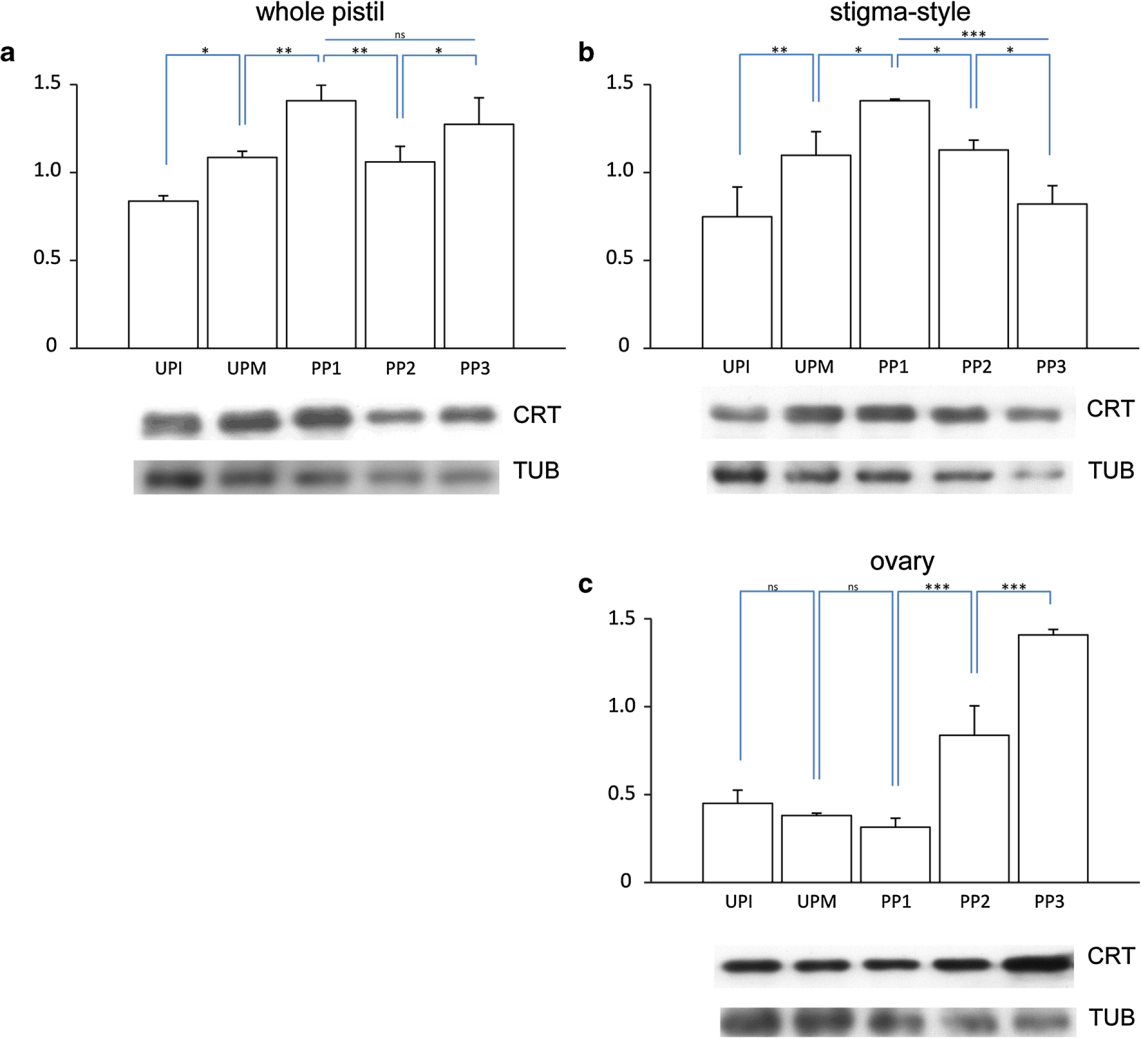
Western blot analysis of *Petunia* CRT levels in whole pistils (**a**), stigma–style fragments (**b**), and ovaries (**c**) before pollination and during subsequent stages of the progamic phase. *Graphs* show the relative CRT levels (mean of three replicates and standard deviation) normalized to the *Petunia* tubulin (TUB) levels. Statistical analysis was carried out by one-way ANOVA (*ns* not significant; *P ≤ 0.05; **P ≤ 0.01; ***P ≤ 0.001). Representative *Western blots* are shown under the *graphs*. *UPI* unpollinated immature pistil, *UPM* unpollinated mature pistil, *PP1*-*PP3* subsequent stages of the progamic phase

If our hypothesis that CRT functions to regulate levels of exchangeable Ca^2+^ in response to anthesis, pollination, and finally fertilization is correct, then we would expect CRT levels to mirror the increase we observed in levels of Ca^2+^ ppts during these processes. To test this, we first performed Western blot analysis of total *Petunia* pistil proteins isolated at the different stages. Using the CRT PAb that we have previously demonstrated to be specific for CRT in *Petunia* pistils (Lenartowska et al. 2002), we detected bands that appear to be doublets (Fig. 6a, b, lines 1–3) or single bands (all other). The CRT PAb likely cross-reacts with the two family members (CRT1 and CRT2 isoforms) that share high sequence homology and functionally compensate each other, and are highly expressed in reproductive tissues (see review by Jia et al. 2009).

Our Western blot experiments showed a gradual increase of the CRT level in whole pistils from the UPI stage to the beginning of the progamic phase and peaking at the PP1 stage (Fig. 6a). A small decrease ~25 % in the CRT level was observed at the PP2 stage, during pollen tube elongation within the pistil, but the level was higher again at the end of the progamic phase, PP3. We speculated that the two peaks of CRT level observed at the PP1 and PP3 stages in whole pistils might reflect increases in CRT level during pollination and fertilization. To assess this possibility, we repeated the immunoblot experiments with protein extracts isolated from the stigma–style and ovary fragments of pistils. In the stigma–style fragments, the levels of CRT expression at the UPI, UPM, and PP1 stages were similar to those observed in the whole pistils (compare Fig. 6a and b). The highest level of CRT expression in the upper part of the pistil was detected at the PP1 stage, when pollen germinated and tubes were outgrowing on the stigma (Fig. 6b). Next, when tubes elongated within the pistil style, the CRT level decreased, reaching its lowest value at the PP3 stage (Fig. 6b). By contrast, CRT levels remained constant in the ovaries at the UPI, UPM, and PP1 stages (Fig. 6c). However, we observed a more than twofold increase in the CRT protein level in the ovary at the PP2 stage, and the level of CRT increased another ~40 % at the PP3 stage, when pollen tubes grow into the ovules resulting fertilization (Fig. 6c). These peaks of CRT protein expression at PP1 in the stigma–style region and at PP3 in the ovary correlate well with our earlier quantitation of *PhCRT* mRNA levels (Lenartowski et al. 2014). Furthermore, these peaks of CRT levels correspond with two key events resulting from pollination: (1) rapid pollen germination and tube outgrowth on the stigma, and (2) pollen tube entry into the embryo sac and fertilization.

### Immunolocalization of CRT in the stigma and ovule at selected stages after pollination

We next sought to determine the localization of CRT in the sitt at the PP1 stage and in the ovule at the PP3 stage, the stages at which our Western blot and Ca^2+^ labeling experiments showed that CRT and exchangeable Ca^2+^ levels changed most dynamically in the particular pistil regions. To do so, we performed immuno-electron microscopy with the CRT PAb and a gold-conjugated secondary antibody.

**Fig. 7:**
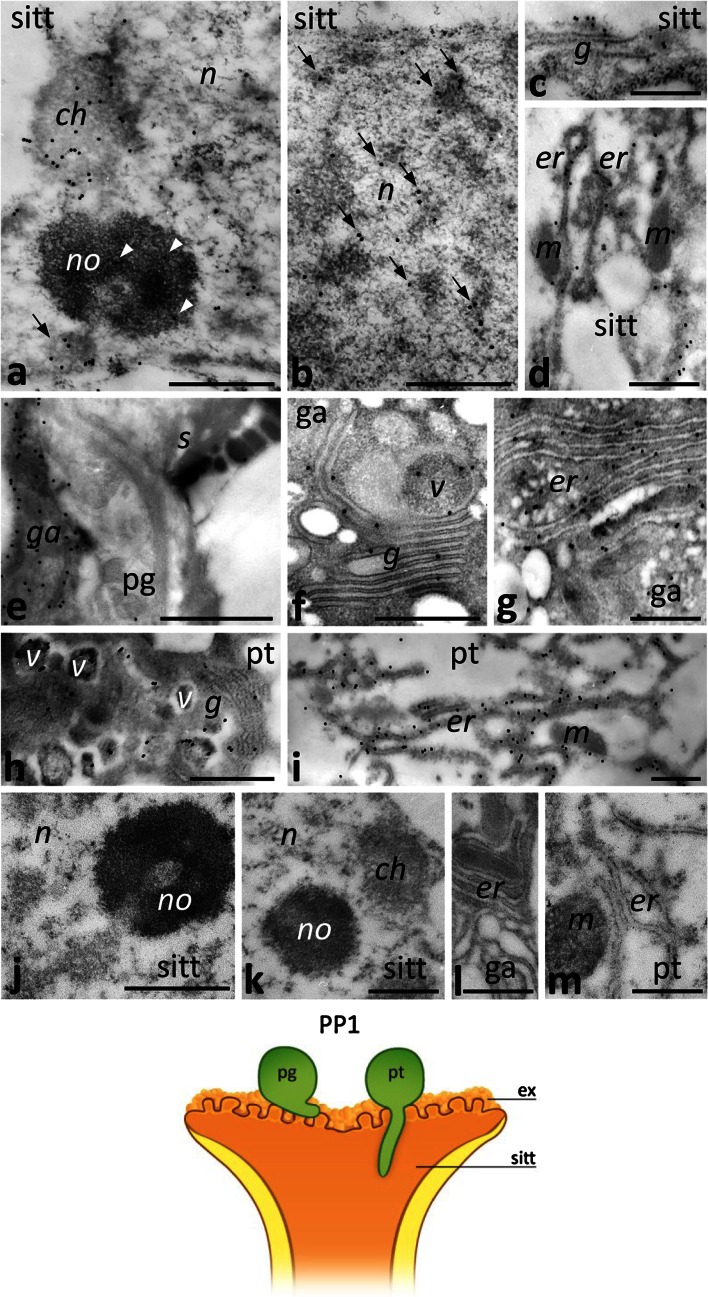
Immunogold localization of CRT in the *Petunia* sitt at the PP1 stage. **a–d** Sitt cells; **e–g** germinated pollen grain (*pg*); **h**, **i** sub-apical zone of the growing pollen tube (*pt*). The control sections in which no CRT PAb was used were devoid of labeling (**j–m**). *Cartoon* shows the analyzed region of the pistil at the PP1 stage. *ch* chromatin, *g* Golgi stack, *ga* germinal aperture, *er* endoplasmic reticulum, *ex* exudate, *m* mitochondrium, *n* nucleus, *no* nucleolus, *s* sporoderm, *v* vesicle. *Bars* 1 μm (**e**), 500 nm (**a**–**d**, **f**–**m**)

Just after pollination, CRT protein was detected in the sitt cell nuclei and cytoplasm. With the immunogold technique, we were able to distinguish CRT localization in defined nucleus sub-domains, such as condensed chromatin (Fig. 7a), perichromatin areas (Fig. 7b, arrows), the nucleolus (Fig. 7a, arrowheads), and the nucleolus-associated chromatin (Fig. 7a, arrow). Within the cytoplasm of sitt cells, CRT was present in the Golgi stacks (Fig. 7c) and in the ER (Fig. 7d). In germinated pollen grains, accumulation of CRT was observed in the germinal aperture (Fig. 7e), where dictyosomes, vesicles, and the ER were evident in the cytoplasm (Fig. 7f, g). A similar CRT labeling pattern was revealed in the cytoplasm of the sub-apical zone of the growing pollen tube. Numerous gold traces were localized in the Golgi stacks, electron-dense vesicles, and the ER (Fig. 7h, i). The control sections in which no CRT PAb was used were devoid of labeling (Fig. 7j–m).

**Fig. 8:**
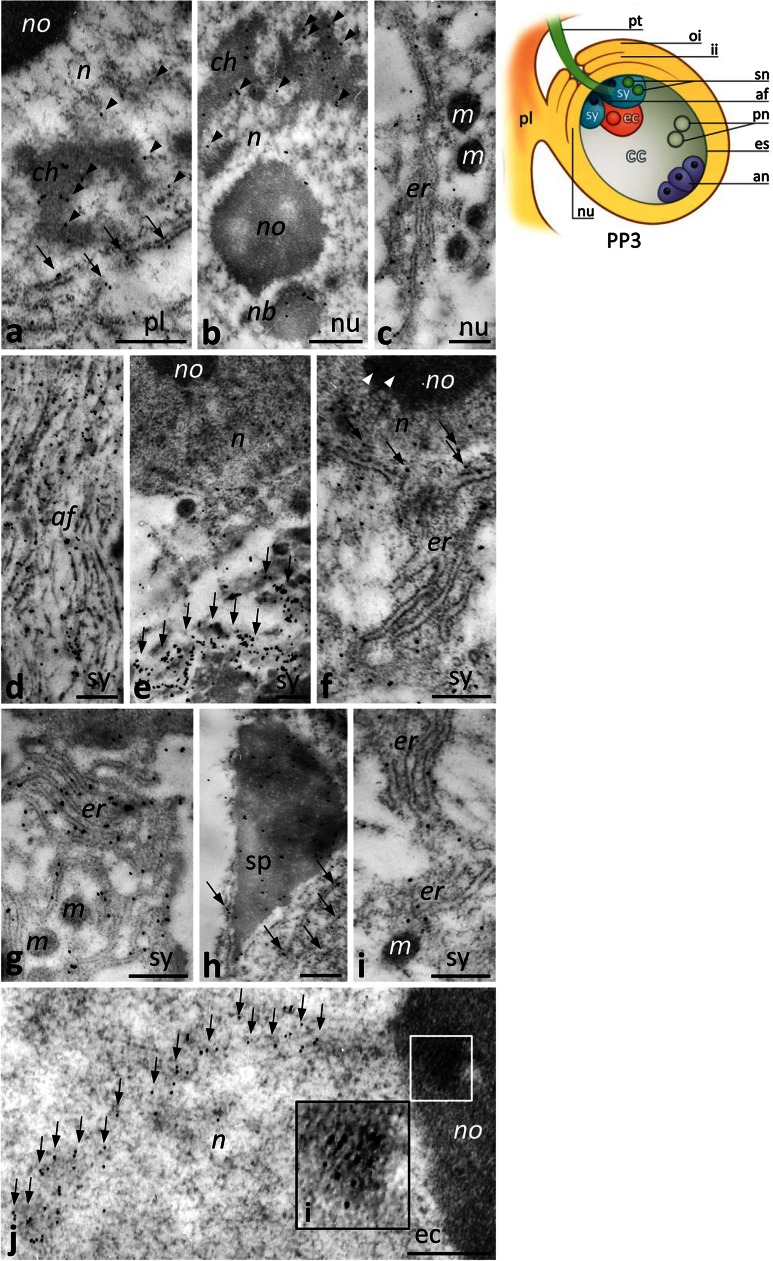
Immunogold localization of CRT in the *Petunia* placental tissue and at the micropylar end of the ovule at the PP3 stage. **a** Placenta tissue (*pl*); **b**, **c** nucellus (*nu*); **d–i** the receptive synergid (*sy*) at the moment of the sperm cells (*sp*) release; **j** the egg cell (*ec*). Inset (**i**) in **j** shows a bigger magnification of the marked region in the *ec* nucleolus (*no*). *Cartoon* shows the ovule at the PP3 stage during sperm cells deposition within the *sy*. *af* filiform apparatus, *an* antipodals, *ch* chromatin, *er* endoplasmic reticulum, *es* embryo sac, *ii* inter integument, *m* mitochondrium, *n* nucleus, *nb* nuclear body, *oi* outer integument, *pn* polar nuclei, *pt* pollen tube, *sn* sperm nucleus. *Bars* 500 nm

At the PP3 stage, CRT labeling was evident in the placenta, the nucellus, and in highly specialized cells of the female gametophyte located at the micropylar pole of the embryo sac, including the receptive synergid and the egg cell (Fig. 8). In the somatic cells of the placenta and the nucellus, CRT was localized in the nuclei (Fig. 8a, b), where it associated with the condensed chromatin, perichromatin areas (Fig. 8a, b, arrowheads), and the nuclear body near the nucleolus (Fig. 8b). Gold traces were found also in the ER (Fig. 8c), including the ER adjacent to the nuclear envelope (Fig. 8a, arrows). The high level of CRT labeling was found in the receptive synergid, particularly within the filiform apparatus, where gold traces were uniformly distributed along the electron-dense fibrils (Fig. 8d), and in the region of the cytoplasm above the site of sperm cells deposition (Fig. 8e, arrows). This cytoplasmic region was extremely rich in the ER cisternae strongly labeled by the CRT antibody (Fig. 8f, g). Some gold traces were also found in the synergid nucleus (Fig. 8e, f), and nucleolus (Fig. 8f, arrowheads) as well as in the nuclear envelope (Fig. 8f, arrows). We observed CRT in the sperm cell deposited within the receptive synergid, both in the sperm nucleus and around the male gamete (Fig. 8h, arrows). At higher magnification, we could discern ER cisternae adjacent to the sperm cell (Fig. 8i). Because the egg cell has very little cytoplasm at the PP3 stage, it was very difficult to clearly identify the organelles labeled by the CRT PAb. However, we found a very specific CRT localization in the egg cell nucleus, where we observed numerous gold traces in the nucleolus (Fig. 8j, marked region and the inset, i) and in a tract within the chromatin (Fig. 8j, arrows).

**Fig. 9:**
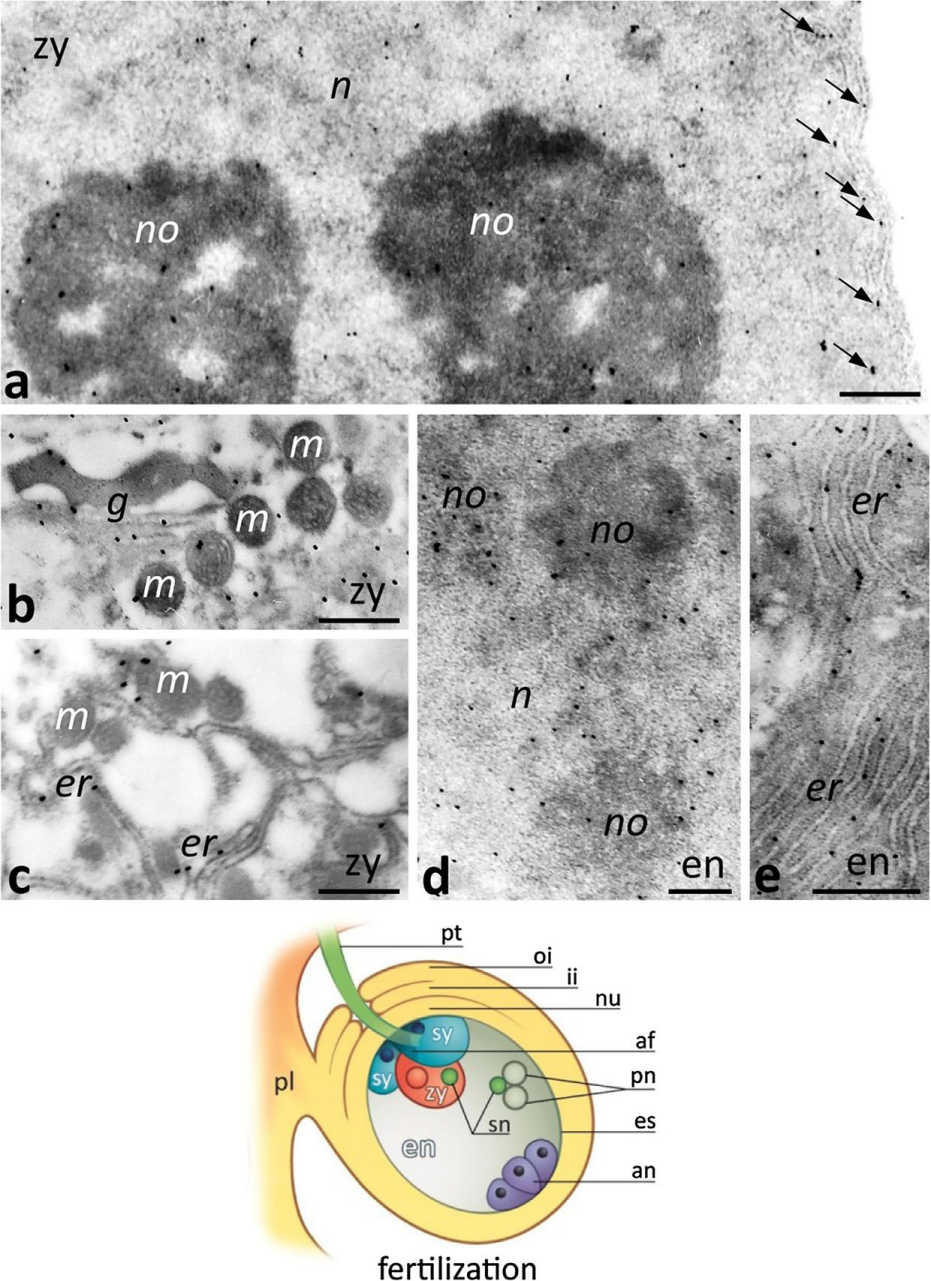
Immunogold localization of CRT in the *Petunia* embryo sac (*es*) at fertilization. **a–c** The diploid zygote (*zy*); **d**, **e** the developing endosperm (*en*). *Cartoon* shows the ovule at fertilization. *af* filiform apparatus, *an* antipodals, *g* Golgi stack, *er* endoplasmic reticulum, *ii* inter integument, *m* mitochondrium, *n* nucleus, *no* nucleolus, *nu* nucellus, *oi* outer integument, *pn* polar nuclei, *pl* placenta, *pt* pollen tube, *sn* sperm nucleus, *sy* synergid. *Bars* 500 nm

After fertilization, CRT labeling was strongest in the zygote (Fig. 9a–c) and in the developing endosperm (Fig. 9d, e). In these cells, CRT was localized in the nucleus, nucleolus, Golgi stacks, and the ER, including the ER adjacent to the nuclear envelope of the zygote (Fig. 9a, arrows).

## Discussion

Here we have demonstrated that both CRT and exchangeable Ca^2+^ have dynamic localization patterns during two key events of plant sexual reproduction: (1) anthesis and pollination resulting in pollen germination and tube outgrowth on the stigma, and (2) pollen tube entry into the receptive synergid, resulting in gamete fusion and embryogenesis.

### CRT is highly expressed in the receptive stigma where pollen germinates and tubes outgrow

This is the first study to fully characterize the sites of exchangeable Ca^2+^ and CRT during the complicated intercellular communication that occurs between the male gametophyte and the female sporophyte/gametophyte cells. Although both external and internal Ca^2+^ stores are likely important in this process, most previous research has focused on the essential role of the ecm in Ca^2+^ storage and mobilization during pollen–pistil interactions. The ER and Golgi stacks are known to be the effective intracellular Ca^2+^ stores (see review by Vandecaetsbeek et al. 2011), and here we provide evidence that these are prominent sites of intracellular Ca^2+^ storage during anthesis, and then pollen germination and tube outgrowth.

We observed that the level of loosely bound Ca^2+^ increased at anthesis in both the sitt ecm and in the cytoplasm of the sitt cells, where Ca^2+^ ppts accumulated mainly in the ER, Golgi stacks, and vesicles. This high level of exchangeable Ca^2+^ was constant until after pollination. Pollen germination and tube growth are known to require Ca^2+^ (see reviews by Ge et al. 2007; Hepler et al. 2012; Dresselhaus and Franklin-Tong 2013; Steinhorst and Kudla 2013). In the pistil, the optimal level of Ca^2+^ required for these process is provided by the pistil transmitting tract. Whereas most studies have focused on Ca^2+^ storage and mobilization from the ecm during pollen–pistil interactions, Iwano et al. (2004) observed that pollination in *Arabidopsis* induces an increase of cytosolic Ca^2+^ in stigma papilla and that this is followed by a gradient of cytosolic Ca^2+^ in hydrated pollen grains. These authors speculated that this pre-germination gradient is generated by the influx of Ca^2+^ from the papillar cells. Although it is not clear whether the Ca^2+^ comes from the cytoplasm or the cell wall of the stigma papilla, our present work in *Petunia* suggests that CRT localized in the ER and Golgi stacks of the sitt cells may provide an exchangeable Ca^2+^ store that can be mobilized at anthesis and during pollination.

The immunoblots revealed a gradual increase in CRT level in the upper part of the pistil from the UPI stage to the beginning of the progamic phase; the highest CRT expression level was at the PP1 stage, when pollen germinates and tubes outgrow on the receptive stigma. During this stage, the immunogold experiments clearly showed that CRT accumulated in the cytoplasm of the germinal pollen aperture and in the cytoplasm of the sub-apical zone of outgrowing tubes, where the labeling was associated with abundant ER cisternae, Golgi stacks, and vesicles. This localization pattern of CRT corresponds well with the high level of loosely bound Ca^2+^ in the same compartments, strongly suggesting that this pool of Ca^2+^ is bound by CRT. It has long been known that growing pollen tubes possess a localized gradient of cytosolic Ca^2+^ at their apex and that an extracellular Ca^2+^ influx occurs at this site (see reviews by Hepler et al. 2012; Dresselhaus and Franklin-Tong 2013; Steinhorst and Kudla 2013). This stable, tip-focused Ca^2+^ gradient is essential for many cellular processes that determine directional pollen tube growth, such as vesicle secretion at the tube tip, cell wall synthesis, tip reorientation, and proper organization and dynamics of the actin cytoskeleton, which differs between the shank and the apical zone of the tube. However, how this Ca^2+^ gradient is sensed and mediated was unknown. We propose that CRT in the ER and Golgi compartments of the pollen tube sub-apical cytoplasm is a key element of the mechanism that allows effective sequestration of free Ca^2+^ from the tube apex. Thus, CRT may play an important role in storing intracellular Ca^2+^ and releasing it when and where it is needed. A key piece of data in support of this idea is our finding that enrichment for exchangeable Ca^2+^ in the ER of the sub-apical zone of the pollen tube was temporary. This finding suggests that the mechanism of modulating local Ca^2+^ concentration within the tube cytoplasm is oscillatory and may correlate with fluctuations in the pollen tube tip-focused Ca^2+^ gradient and other growth oscillations of the pollen tube. CRT likely plays an analogous role during pollen tube directional elongation to the ovary because we have previously observed similar distributions of CRT and exchangeable Ca^2+^ in the pollen tubes and sytt cells within the *Petunia* and *Haemanthus* pistils (Lenartowska et al. 1997, 2002, 2009).

We previously found that the highest level of *PhCRT* mRNA expression was in the stigma–style fragment of UPI pistil (Lenartowski et al. 2014). In addition, we observed a higher level of *PhCRT* transcripts in the sitt cells before anthesis than in UPM pistil. Together, these results indicate that *PhCRT* transcription in sitt cells starts as early as the flower bud stage, whereas CRT translation is postponed and reaches maximum level at anthesis and pollination. During anthesis, the exudate fills intercellular spaces between the sitt cells and covers the receptive *Petunia* stigma. The stigma thus becomes wet due to the high secretory activity of these specialized cells. High levels of CRT have been observed in other plant tissues that are secretory in nature, such as the aleurone layer of the barley caryopsis (Dedhar 1994), nectaries of *Arabidopsis* flowers (Nelson et al. 1997), endothelium of the integument in *Nicotiana* fertilized ovules (Borisjuk et al. 1998), and the active tapetum of *Nicotiana* anthers (Nardi et al. 2006). Secretory cells are characterized by very high rates of protein synthesis, the ER cisternae, and numerous dictyosomes in close association with the plasma membrane. We have observed similar ultrastructure in the *Petunia* sitt cells, in which CRT was highly abundant. Our immunogold experiments further revealed that CRT preferentially localizes to the ER and Golgi stacks of sitt cells during the PP1 stage. Thus, we suggest that in addition to regulating Ca^2+^ homeostasis, the high level of CRT expression in the *Petunia* sitt reflects a role for CRT as a chaperone to achieve high secretory activity of the sitt specialized cells to ensure the stigma receptivity.

### The highest levels of exchangeable Ca^2+^ and CRT in the ovary correspond with pollen tube entry into the embryo sac and double fertilization

Pollen tube extends through the pistil transmitting tract and is guided to ovule where it releases two sperm cells in the receptive synergid for fertilization. Our previous (Lenartowski et al. 2014) and present reports support the hypothesis that CRT is required to maintain Ca^2+^ homeostasis during both restricted sperm cell release and gamete fusion. First, the Western blot analysis showed that the highest level of CRT expression correspond with these events. Second, we observed a dramatic increase in the level of loosely bound Ca^2+^ at the micropylar end of the ovule where pollen tube penetration and fertilization occur. We found numerous Ca^2+^ ppts in the ER and dictyosomes of somatic placental and nucellus cells as well as in the female gametophyte. Third, we saw a large mass of Ca^2+^ ppts in the ER and Golgi stacks of the receptive synergid during sperm cells deposition and in the zygote and developing endosperm. Finally, we previously reported accumulation of *PhCRT* mRNA within the receptive synergid, the zygote, and the endosperm (Lenartowski et al. 2014). Our results are consistent with other reports that confirm up-regulation of CRT expression at fertilization or during embryo development in other plant species (Chen et al. 1994; Dresselhaus et al. 1996; Nelson et al. 1997; Borisjuk et al. 1998). These authors speculated that CRT is required for rapid cell divisions of the zygote and early endosperm or for proper folding of the proteins that are actively synthesized by the developing embryo and nutritive tissue. Recent beautiful experiments in *Arabidopsis* have shown that the CRT3 isoform, together with another ER luminal protein, POD1, regulate the pollen tube response to female gametophyte signals and cell plate patterning during early embryogenesis (Li et al. 2011). These authors speculated that POD1, via modulating CRT3 function, plays a key role in the ER quality control system and/or modulates cytosolic Ca^2+^ homeostasis and its regional concentration by tethering CRT3. However, unlike the highly acidic C-domains in the *Arabidopsis* CRT1/2 isoforms and their mammalian counterpart CRT1, the C-domain of the *Arabidopsis* CRT3 isoform does not have high-capacity Ca^2+^-binding ability (Qiu et al. 2012b). We previously demonstrated that the *PhCRT* that is expressed in *Petunia* pistil belongs to the *CRT1/CRT2* subclass (Lenartowski et al. 2014); this class generally functions as ER chaperones involved in protein folding and Ca^2+^ homeostasis (see review by Jia et al. 2009). Taken together, our previous and present research suggest that the CRT protein expressed in the *Petunia* ovule functions mainly as a mobile Ca^2+^ store and regulator of Ca^2+^ homeostasis during the last stage of the progamic phase and at fertilization. This idea is consistent with the fact that both gamete fusion and fertilized egg activation are Ca^2+^-related processes (see review by Ge et al. 2007). We do not exclude that CRT highly expressed during fertilization and early embryogenesis may also function as the ER chaperone as other authors suggest.

One of our most interesting observations was that both CRT and exchangeable Ca^2+^ were concentrated within the filiform apparatus and the cytoplasm of the receptive synergid, above the site of sperm cells deposition. CRT and exchangeable Ca^2+^ were localized mainly in the ER, which is prominent in the synergid cytoplasm and surrounding the sperm cells. Our previous work likewise revealed that the highest concentration of *PhCRT* mRNA was in this same cytoplasmic region of the receptive synergid (Lenartowski et al. 2014). These findings are consistent with reports that, of all the cells in the embryo sac, the highest level of loosely bound Ca^2+^ is in the receptive synergid (see review by Ge et al. 2007). Furthermore, a dramatic increase of cytoplasmic Ca^2+^ occurs in the receptive synergid at pollen tube rupture in *Arabidopsis* (Iwano et al. 2012). It is still unknown whether the Ca^2+^ is delivered from the ecm, the ER, or both. Several functions have been proposed for the filiform apparatus, including pollen tube reception, import of metabolites, and export of the pollen tube attractants, and numerous proteins have been found to localize in this highly specialized compartment (see review by Dresselhaus and Franklin-Tong 2013). We postulate that CRT may provide an exchangeable Ca^2+^ store in the receptive synergid and modulate the local concentration of Ca^2+^ to prevent extra pollen tubes from entering the embryo sac. Interestingly, we observed large patches of Ca^2+^ ppts above the released sperm cells within the receptive synergid. We also found the ER cisternae enriched with CRT in this same location. These findings are consistent with a report of CRT patches in *Nicotiana*; these were interpreted to serve as a specific Ca^2+^ storage mechanism (Borisjuk et al. 1998). Alternatively, CRT may be involved in regulation of Ca^2+^ homeostasis during positioning of the male gametes for fusion with the female gametes. The two released sperm cells migrate to fertilize the egg cell and the central cell, and this movement of non-motile male gametes within the embryo sac appears to depend on actin-myosin interactions (Fu et al. 2000; Ye et al. 2002). Actin dynamics is a Ca^2+^-dependent process, and Ca^2+^ regulates the activities of many actin-binding proteins.

Although the precise mechanism is unclear, maintenance of an optimal Ca^2+^ environment within the female gametophyte is essential for fertilization. CRT is an excellent candidate to fulfill this role. We proposed this idea previously when we observed a micropylar-chalazal gradient of *PhCRT* mRNA in the receptive synergid (Lenartowski et al. 2014). Our findings here support this hypothesis because we found long ER cisternae surrounding the sperm cells in the synergid, and this structure was enriched with CRT and exchangeable Ca^2+^. Additionally, we have detected both CRT and Ca^2+^ in *Petunia* sperm cells released within the receptive synergid. In animals, the CRT2 isoform is highly expressed in testes (Persson et al. 2002), and CRT is localized with spherical spermatids and mature sperm (Nakamura et al. 1993; Naaby-Hansen et al. 2001; Ho and Suarez 2003). CRT and Ca^2+^ oscillations have also been reported in maize sperm cells (Williams et al. 1997a, b), and CRT localizes to spermatids in the higher alga *Chara* (Popłońska 2013). Together, these results strongly suggest that CRT serves unique functions within the male gametes, but their determination requires further study.

### Potential role of CRT in the nucleus

Here we report the presence of CRT in the nuclei of several *Petunia* pistil sporophytic and gametophytic cells such as secretory sitt cells, somatic cells of the placenta and nucellus, the haploid receptive synergid, the egg cell, the zygote, and the triploid endosperm. In these cells, CRT localized to the nuclear envelope and to different nuclear sub-domains. CRT has been suggested to have a function in the nucleus owing to its putative nuclear localization signal (NLS; see review by Michalak et al. 1992). Additionally, immunofluorescence studies have revealed CRT in the nuclear envelope, nuclear matrix, and nucleolus in some animal cells (see reviews by Michalak et al. 1992, 2009; Gelebart et al. 2005). Nuclear-localized CRT has been implicated in steroid-sensitive gene expression, export of steroid hormone receptors to the cytoplasm, and modulation of stability of certain mRNAs. In addition, CRT has been detected in association with histones in mitotic chromosomes (Kobayashi et al. 2006) and with peripheral chromatin in the nucleus of the protozoan *Trypanosoma* (Souto-Padrón et al. 2004). Given the presence of Ca^2+^ on the axis of metaphase chromosomes (Strick et al. 2001) and the high Ca^2+^ storage capacity of CRT, these reports suggest that CRT stores exchangeable Ca^2+^ to supply Ca^2+^-dependent scaffold proteins that regulate the dynamics of chromatin fibers.

Plant CRT has been also found in the nuclear envelope (Denecke et al. 1995; Napier et al. 1995) and within the nucleus (Lenartowska et al. 2002). Using a transient expression assay, Jia et al. (2008) demonstrated that a wheat CRT3 isoform coupled to green fluorescent protein (GFP) localized to both the nucleus and cytosol of living onion epidermal cells. Moreover, during spermiogenesis in the green alga *Chara*, CRT co-localized with protamine-type proteins within the spermatid nucleus, on chromatin, and in the extensive system of ER cisternae and vesicles connected to the nuclear envelope (Popłońska 2013). The author suggested that CRT participates in the exchange of nuclear proteins that allow for extreme chromatin condensation during spermatogenesis. Our present research showed that CRT localized within chromatin, including the condensed chromatin, the perichromatin areas, the nucleolus, nucleolus-associated chromatin, and nuclear bodies (probably Cajal bodies) of several *Petunia* pistil cell types. Several theories could explain the functional importance of CRT within the nucleus. Perichromatin fibrils are the sites of transcription and co-transcriptional processing of pre-mRNA and maturation of pre-RNA/mRNA (see review by Biggiogera et al. 2008), and Cajal bodies are important elements of the mRNA splicing system (see review by Shaw and Brown 2004). Therefore, CRT associated with these nuclear sub-domains may be involved in regulating a large spectrum of nuclear processes. Additionally, CRT may play a role in ribosome biogenesis or other functions of the nucleolus (see review by Shaw and Brown 2011). In the egg cell nucleus, we observed CRT localization in the nucleolus and as a distinguishable tract within the chromatin. Thus, CRT may be involved in nuclear/nucleolar transport of macromolecules.

We found that CRT’s localization in the nuclear envelope and within the nuclei of different *Petunia* pistil cell types corresponded with sites of exchangeable Ca^2+^. This could indicate that CRT modulates nuclear Ca^2+^ homeostasis by regulating the local Ca^2+^ concentration. The lumen of the nuclear envelope is contiguous with the lumen of the ER and can form a branching intra-nuclear network (see review by Gomes et al. 2006). This structure, called the nucleoplasmic reticulum (NR) in animals, can occasionally be observed in plants. Like the ER, the NR could serve as a mobile store of Ca^2+^ (see review by Mazars et al. 2011), which has been shown to play a role in transcriptional regulation in plants (see review by Ranty et al. 2012). This idea is strengthened by the fact that some components necessary for inositol-1,4,5-triphosphate (InsP_3_)-mediated Ca^2+^ signaling were found in both the nuclear envelope and the NR of animal cells. Existence of such Ca^2+^-signaling machinery has also been confirmed in purified tobacco nuclei. InsP_3_ induces release of Ca^2+^ from both the nuclear envelope and the NR into the nucleoplasm, resulting in generation of physiologically important Ca^2+^ signals that can be recognized by Ca^2+^-binding proteins involved in signaling. This is also a mechanism of maintaining nuclear Ca^2+^ homeostasis (see reviews by Gomes et al. 2006; Ranty et al. 2012).

Nuclear localization might be unexpected given that plant CRT has typical ER targeting and retention (HDEL) signals and is usually found primarily in the ER. However, the rough ER is continuous with the outer nuclear membrane, and some authors suggest a possible function of CRT in the lumen of the ER/nuclear envelope rather than in the nucleoplasm. By contrast, our present immunogold research shows, for the first time, highly selective CRT localization in specific nuclear sub-domains. How CRT could leave the ER and move into the nucleus remains unknown. Possible explanations include production of splice variants that can localize to both places, posttranslational modifications such as glycosylation, degradation of the C-domain containing HDEL, or enzymatic modification of the glycan complexity (see reviews by Johnson et al. 2001; Michalak et al. 2009). Experiments by Brandizzi et al. (2003) suggest that CRT can be actively transported to the nucleus on microtubules. They observed that misfolded GFP-CRT (different subunits of CRT) was retrogradely transported from the ER lumen to the cytosol and nucleoplasm in plants. CRT may also enter the nucleus as a protein complex. For example, Sharma et al. (2004) characterized a nuclear protein (CRTintP) that strongly interacts with CRT in rice, contains an NLS, and localizes to the nuclei of onion epidermal cells. Furthermore, co-immunoprecipitation confirmed the existence of a CRT-CRTintP complex in vivo in stressed leaf tissue, suggesting a role for these proteins in controlling stress responsive genes. Despite these findings, it still remains unclear why CRT is not universally observed in the nucleus, and further investigations are needed to explain its roles in this compartment.

In conclusion, our results suggest that the precise regulation of Ca^2+^ homeostasis during anthesis and pollen–pistil interactions in plants depends on intracellular Ca^2+^ stores and CRT. CRT’s Ca^2+^-binding/buffering property is probably essential to control mobilization of stored Ca^2+^ from the ER, Golgi compartments, and nuclei. We consider the possibility that CRT enters nuclei only during certain events, such as generative reproduction in plants. Additionally, CRT’s role as a molecular chaperone may be equally important because a high rate of protein synthesis is required during the multi-step process of plant sexual reproduction. Future functional work will help us sort out these possibilities.

## Abbreviations

Ca^2+^ ppts: Calcium-antimonate precipitates
*CRT*: Calreticulin gene
CRT: Calreticulin protein
CRT PAb: Polyclonal antibody against CRT
Ecm: Extracellular matrix
GFP: Green fluorescent protein
HDEL: ER-retention sequence of plant CRT
NLS: Nuclear localization signal
NR: Nuclear reticulum
PP1: First stage of the progamic phase
PP2: Second stage of the progamic phase
PP3: Third stage of the progamic stage
*PhCRT*: *Petunia hybrida* calreticulin gene
Sitt: Stigma transmitting tract
Sytt: Style transmitting tract
UPI: Unpollinated immature pistil
UPM: Unpollinated mature pistil
